# Effect of sacubitril valsartan on heart failure with mid-range or preserved ejection fraction in patients on maintenance hemodialysis: real-world experience in a single-center, prospective study

**DOI:** 10.1186/s12872-024-03744-y

**Published:** 2024-01-30

**Authors:** Xiao-mei Huang, Jing-jing Li, Wang Yin, Hui-ling Fu, Fen Yu, Lian-qing Gu, Yi Zhang, Min Du, Zheng Ye, Li Xu

**Affiliations:** 1 Department of Nephrology, Tongji Medical College, The Central Hospital of Wuhan, Huazhong University of Science and Technology, Wuhan, 430014 China; 2 Department of Ultrasound, Tongji Medical College, The Central Hospital of Wuhan, Huazhong University of Science and Technology, Wuhan, 430014 China; 3 Department of Public Health, Tongji Medical College, The Central Hospital of Wuhan, Huazhong University of Science and Technology, Wuhan, 430014 China

**Keywords:** Sacubitril valsartan, Heart failure with preserved ejection fraction, Heart failure with mid-range ejection fraction, Mortality, Hemodialysis, Pulmonary hypertension

## Abstract

**Background:**

This study aimed to evaluate the effect of sacubitril valsartan (SV) on heart failure (HF) hospitalization and cardiovascular mortality in patients on hemodialysis with HF with preserved ejection fraction (EF; HFpEF).

**Methods:**

This single-center, prospective study enrolled 155 stable hemodialysis patients with EF > 40% who were followed up for 12 months. Fifty-nine patients were treated with SV; the others were matched for EF (57.89 ± 9.35 vs. 58.00 ± 11.82, *P* = 0.9) at a ratio of 1:1 and included as controls. The target dosage of SV was 200 mg/day.

**Results:**

Twenty-three (23/155; 14.84%) had HF with mid-range EF (HFmrEF), while 132 (85.16%) had HFpEF. After SV treatment, the peak early diastolic transmitral flow velocity/peak early diastolic mitral annular tissue velocity(E/e’) improved from 17.19 ± 8.74 to 12.80 ± 5.52 (*P* = 0.006), the left ventricular (LV) end-diastolic diameter decreased from 53.14 ± 7.67 mm to 51.56 ± 7.44 mm (*P* = 0.03), and the LV mass index decreased from 165.7 ± 44.6 g/m^2^ to 154.8 ± 24.0 g/m^2^ (*P* = 0.02). LVEF (*P* = 0.08) and LV global longitudinal strain (*P* = 0.7) did not change significantly. The composite outcome of first and recurrent HF hospitalization or cardiovascular death showed no difference between group. However, the Acute Dialysis Quality Initiative Workgroup (ADQI) HF class improved in 39 and 15 patients and worsened in 1 and 11 patients in the SV and control groups, respectively (*P* < 0.001). Age, diabetes mellitus, and pulmonary arterial pressure were independent risk factors for HF hospitalization and cardiovascular mortality in patients with HFpEF.

**Conclusions:**

SV improved LV hypertrophy, diastolic function, and the ADQI class for HF; however, it failed to reduce the composite endpoints of HF hospitalization and cardiovascular disease-related mortality over 12 months of follow-up in patients on maintenance hemodialysis with EF of > 40%.

## Background

Several clinical studies have suggested that sacubitril valsartan (SV) can reduce the rates of heart failure (HF) hospitalization and cardiovascular disease-related mortality in patients with chronic HF with ejection fractions (EFs) of ≤ 40% [1, 2]. However, HF with mid-range ejection fraction (HFmrEF) and HF with preserved ejection fraction (HFpEF) are very common in clinical practice and are associated with high rates of HF hospitalization and cardiovascular disease-related mortality [3, 4], and effective treatments have not been established [5]. The PARAGON-HF [6] study reported that patients with HF and EFs of 45–57% and female patients may benefit from SV, although it does not significantly reduce the rates of HF hospitalization and cardiovascular disease-related mortality. However, patients with moderate-to-severe chronic kidney disease were not included in the study.

In recent years, SV has been used for patients with moderate-to-severe chronic kidney disease, including patients on maintenance hemodialysis (MHD) [7–9], and is reported to improve renal function and facilitate effective blood pressure control with a manageable risk of hyperkalemia and severe hypotension. Studies on cardiovascular events and HF hospitalization of patients on MHD are limited. Unlike in non-dialysis patients, the cardiovascular system in patients on MHD is affected by uremic toxins, volume loads during the inter-dialysis period, dialysis ultrafiltration, and vascular access. The prevalence of HF among patients on MHD ranges from 40 to 76.5% [10, 11]. HFpEF is a common cardiovascular disease in patients on MHD, and survival is significantly reduced in patients on MHD with HF. Based on the clinical heterogeneity of the cardiac phenotype in patients with HFpEF [12, 13] and the special characteristics of the dialysis patients, we aimed to investigate the effect of SV on the rates of HF hospitalization and cardiovascular disease-related mortality among patients on MHD with EF of ≥ 40%.

## Methods

### Population

This was a single-center, real-world, prospective study. Patients receiving MHD in Wuhan Central Hospital between July 2021 and July 2022 were screened. This study was approved by the ethics committee of the Wuhan Central Hospital (approval Document: 2016 Medical Research No. 03 and Hospital-Heng-Lun letter-2021 (9)). Informed consent was obtained from each patient, and the study protocol conformed to the ethical guidelines of the 1975 Declaration of Helsinki.

The inclusion criteria were as follows: (a) blood pressure > 100/60 mmHg; (b) age > 18 years and expected survival duration of ≥ 1 year; (c) hemodialysis vintage ≥ 12 months and willingness to participate in the study; (d) symptoms of HF, including fatigue, edema, dyspnea, and pulmonary or systemic congestion; (e) Acute Dialysis Quality Initiative (ADQI) heart functional class for HF from 2R to 4NR in dialysis patients [14]; (f) fulfillment of criteria for the diagnosis of HF with documented EF > 40% based echocardiography within 6 months before screening [15, 16].

The exclusion criteria were as follows: (a) refusal to heed medical advice or loss to follow-up; (b) prior EF of < 40% detected by echocardiography; (c) HF primarily resulting from precordial hypertrophic obstructive cardiomyopathy, severe heart valve disease (mitral valve lesion/aortic valve lesion), isolated right HF, constrictive pericarditis, and cardiac resynchronization treatment combined with malignancy, congenital heart disease, or tuberculosis; (d) weight gain of ≥ 10% of dry weight between dialysis sessions, even with a dialysis frequency of 3 times per week and total dialysis time of ≥ 10 h/week (this condition occurred repeatedly more than 3 times within 1 month of screening [17]); and (e) allergy to angiotensin receptor antagonists or enkephalinase inhibitors.

**Fig. 1:**
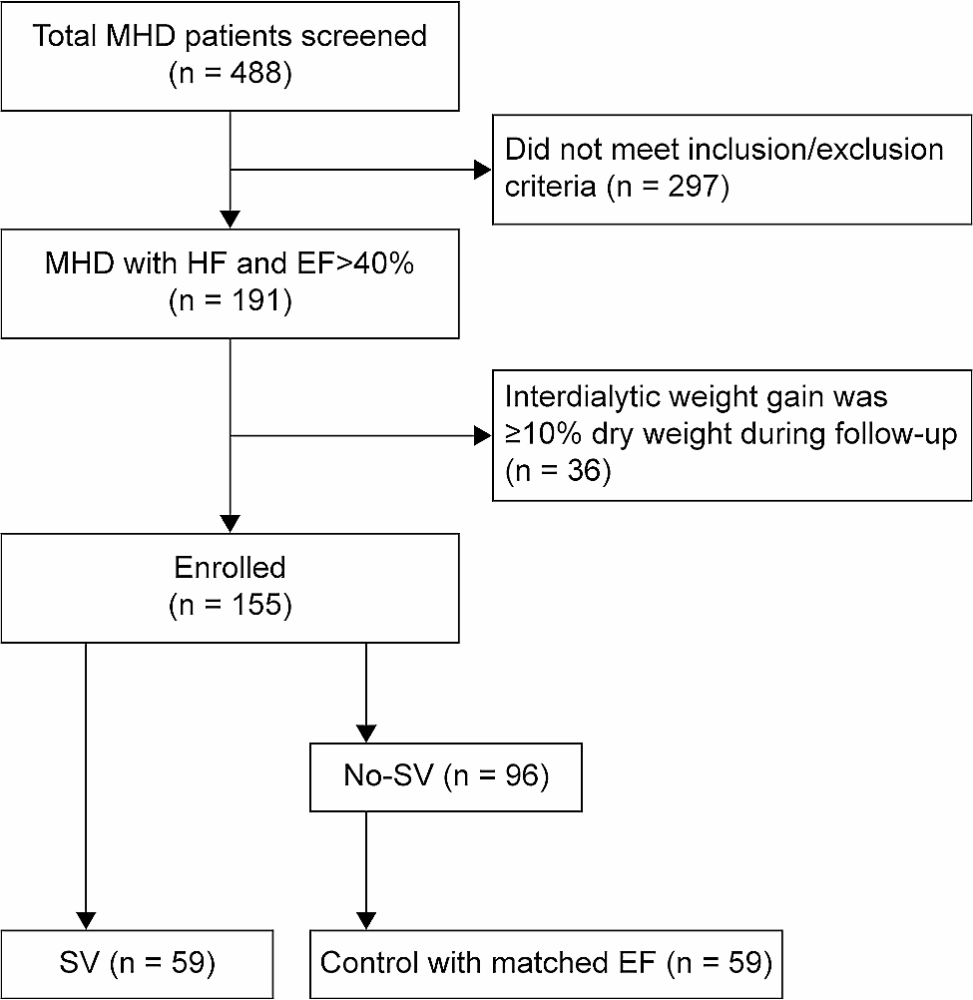
Study flow chart Abbreviations: MHD, maintenance hemodialysis; HF, heart failure; EF, ejection fraction; SV, sacubitril valsartan

All patients received regular hemodialysis or hemodiafiltration 3 times/week, with each session lasting for 4 h. SV (Beijing Novartis Pharmaceutical Co., LTD., national medicine standard J20190002) was administered to 59 (SV group) of the 155 eligible patients. The other 96 patients did not use SV (non-SV). In prior analyses, EF in the non-SV group was better than that in the SV group. Therefore, in our study, controls were selected from the 96 non-SV patients with matched EF at a 1:1 ratio (Fig. 1).

The SV dosage was 25–100 mg, administered orally twice a day. The target dosage of SV was 200 mg/day (sacubitril 97 mg, valsartan 103 mg). At the beginning of SV treatment, the original antihypertensive drugs were changed to SV; the angiotensin-converting enzyme inhibitors/angiotensin receptor antagonists in the original antihypertensive regimens were changed to SV, or SV was added to the original antihypertensive medicines to control blood pressure. If systolic blood pressure of ≤ 100 mmHg or diastolic blood pressure of ≤ 60 mmHg persisted for more than 2 days, the dry weight of patients on MHD was re-estimated, and the doses of the antihypertensive drugs, except SV, were reduced until discontinuation before considering the reduction of the dose of SV. Other treatments, such as those for the management of renal anemia or control of chronic kidney disease and mineral/bone disorders, were regularly administered.

The estimated glomerular filtration rate was calculated based on the Chronic Kidney Disease Epidemiology Collaboration equation [18].

### Echocardiography methods

The Philips IE Elite color Doppler ultrasound with S5-1 phased-array transducer (1-5 MHz frequency range, Philips Medical Systems, Bothell, WA, USA) was used for echocardiographic examinations. To prevent the effects of pre-dialysis water and sodium retention, echocardiographic examinations of patients on MHD were performed 24 h after hemodialysis completion. The cardiac sonographers did not know whether the patients used SV.

Conventional echocardiographic parameters were measured according to the ultrasound measurement method recommended by the guidelines [15, 16]. All measurements were performed in 3 consecutive cycles, and the mean values were calculated. In brief, the left ventricular (LV) mass index (LVMI) was calculated as the LV mass divided by the body surface area. Left atrial volume, LV ejection fraction (EF), and cardiac output were determined using Simpson’s biplane method. The peak velocity of longitudinal contraction (s’) and early diastolic peak velocity (e’) were measured using tissue Doppler in the four-chamber view. The tricuspid annular plane systolic excursion (TAPSE) was measured using M-mode tracing from the apical four-chamber view. Tricuspid regurgitation was measured using a four-chamber view. Systolic pulmonary artery pressure (PAsP) was calculated from the tricuspid valve pressure gradient and the central venous pressure (based on the collapse rate of the inferior vena cava during respiration and the internal diameter of the inferior vena cava).

Myocardial strain was calculated using speckle tracking from two-dimensional grayscale images. The recordings were stored as raw data and analyzed offline by two experienced echocardiographic physicians using Philips QLAB software (Philips Healthcare, The Netherlands) blinded to the clinical information.

### Identification of cardiovascular events

Cardiovascular events were considered as acute and chronic HF, cardiac arrest, or sudden death in this study. The cardiovascular events that occurred and the cause of death were recorded during the follow-up. When patients died out-of-hospital, their families were interviewed by telephone for possible causes of death. When patients had multiple cardiovascular events, the time of the first event was used for Cox analysis. Patients converted to renal transplantation or peritoneal dialysis were recorded as censored.

### Endpoints

The primary endpoints included the composite of first HF hospitalization or cardiovascular disease-related mortality and the composite of the total number of (first and recurrent) HF hospitalizations and cardiovascular disease-related deaths.

The secondary endpoints included changes in heart function evaluated by the ADQI class for HF in patients on MHD at baseline and 12 months later and all-cause mortality.

### Adverse events

Severe hypotension (blood pressure ≤ 90/60 mmHg), angioedema, liver dysfunction, and severe hyperkalemia (blood potassium ≥ 6.5 mmol/L detected more than 2 times) that were not corrected by adequate dialysis, dietary guidance, and medications were considered as adverse events based on the judgment of 3 physicians unless uremia or dialysis-related co-morbidities were excluded.

### Statistical analysis

All statistical analyses were conducted using SPSS 23.0 statistical software (IBM Corp., Chicago, IL, USA). The normally distributed data were summarized as mean ± standard deviation, and non-normally distributed continuous data were reported as median and interquartile ranges. The *t*-test and Wilcoxon test were used for the comparison of continuous data, and the *χ*^2^ or Fisher’s exact test was used for the comparison of categorical data. The relationship between echocardiographic parameters and HF hospitalization was analyzed by Cox regression analysis. A *P*-value of < 0.05 denoted statistical significance.

## Results

### Baseline clinical characteristics of patients on MHD

The baseline clinical characteristics of patients on MHD are presented in Table 1. Both groups of participants in this study were matched by LVEF at a 1:1 ratio. Their baseline data, including diseases, ADQI class for HF, hemodialysis vintage, average ultrafiltration volume per dialysis session in a week, laboratory tests, and antihypertensive medicines, were comparable.

**Table 1:** Baseline clinical characteristics of maintenance hemodialysis patients stratified into sacubitril valsartan and control groups

	SV (*n* = 59)	Control (*n* = 59)	*p*/*x*^2^
Age (years)	59.94 ± 12.64	58.81 ± 12.31	0.9
Sex, n (%)			0.2
Male Female	41 (69.49) 18 (30.51)	51 (57.89) 24 (42.11)	-- --
BMI SBP DBP	22.77 ± 3.27 151.71 ± 21.21 81.71 ± 13.31	23.94 ± 4.96 145.72 ± 20.17 81.30 ± 13.38	0.07 0.2 0.8
ADQI class for HF - n (%)			0.2
2R-2NR 3R-3NR 4R-4NR	35 (59.32) 16 (27.12) 8 (13.56)	42 (72.41) 16 (27.59) 0	-- -- --
Baseline disease-n (%)			
Hypertension Diabetes	52 (88.13) 23 (38.98)	51 (87.93) 22 (37.93)	0.9 0.9
HD vintage (months)	52 (24, 63)	55 (27, 83)	0.4
Smoking history-n (%)	32 (54.23)	24 (40.68)	0.2
UF (L)	2.74 ± 0.76	2.91 ± 0.85	0.2
Scr (umol/L)	881.84 ± 245.38	876.09 ± 245.38	0.9
BUN	21.96 ± 6.29	22.29 ± 6.30	0.8
eGFR (CKD-EPI)	4.96 ± 0.89	4.75 ± 1.01	0.8
Alb (g/L)	40.14 ± 1.66	4.56 ± 4.09	0.6
K (mmol/L))	4.98 ± 0.71	4.99 ± 0.72	0.9
Na (mmol/L)	140.20 ± 3.55	141.30 ± 3.87	0.9
Cl (mmol/L)	103.70 ± 8.72	105.10± 8.46	0.7
Ca (mmol/L)	2.29 ± 0.16	2.32 ± 0.20	0.4
*P* (mmol/L)	1.75 ± 0.50	1.86 ± 0.52	0.2
hs-CRP (mg/L)	2.00 (1.00-3.40)	1.80 (1.00–3.60)	0.5
LDL (mmol/L)	1.98 ± 0.75	2.26 ± 0.76	0.7
TC (mmol/L)	3.56 ± 0.90	3.90 ± 1.09	0.2
Antihypertensive medicines – n (%)			0.5
RASI	40 (67.80)	41 (70.83)	
BB CCB αB	35 (59.32) 47 (79.66) 17 (28.81)	30 (51.04) 47 (81.25) 10 (16.67)	-- -- --

Values are expressed as n, mean ± SD, n (%), or median (interquartile range), unless otherwise indicated

Abbreviations: SV, sacubitril valsartan; BMI, body mass index; SBP, systolic blood pressure; DBP, diastolic blood pressure; ADQI, The Acute Dialysis Quality Initiative Workgroup; HF, heart failure; HD, hemodialysis; UF, ultrafiltration; Scr, serum; BUN, blood urea nitrogen; eGFR, estimated glomerular filtration rate, CKD-EPI, chronic kidney disease collaboration equation; Hb, hemoglobin; Alb, albumin; K, Kalium; Ca, calcium; P, phosphorus; RASI, renin-angiotensin-aldosterone system inhibitor; BB, β-receptor blocker; CCB, calcium channel blocker; αB, α-receptor blocker; LDL, low-density lipoprotein cholesterol; TC, total cholesterol; hs-CRP, high sensitivity C-reactive protein

### Echocardiography at baseline in both groups

Twenty-three (14.84%) of the 155 enrolled patients had HFmrEF, while 132 (85.16%) had HFpEF. After matching by LVEF at a 1:1 ratio, the LVEF distribution and level showed no significant between-group difference (57.89 ± 9.35 vs. 58.00 ± 11.82, *P* = 0.9). However, several left heart parameters were worse for the SV group than for the control group; these included the LV end-diastolic diameter (LVEDd, 53.14 ± 7.67 vs. 49.45 ± 7.54, *P* = 0.01), the LV global longitudinal strain (LVGLS, -14.87 ± 4.40 vs. -16.91 ± 2.64, *P* = 0.03), the LVMI (165.70 ± 44.63 vs. 138.18 ± 44.69, *P* < 0.001), and peak early diastolic transmitral flow velocity/peak early diastolic mitral annular tissue velocity (E/e’; 17.19 ± 8.74 vs. 12.31 ± 6.42, *P* = 0.002). Echocardiographic parameters for right heart function, such as TAPSE, the fractional area change (FAC), the right ventricular (RV) myocardial work index (RIMP), the RV global longitudinal strain (RVGLS), PAsP, TAPSE/PAsP, and RVGLS/PAsP, showed no significant between-group differences. PAsP was above 30 mmHg in more than 50% of patients on MHD in both groups (31 in the SV group and 30 in the control group) (Table 2).

**Table 2:** Echocardiography results at baseline for the sacubitril valsartan and control groups

	SV *N* = 59	Abnormal	control *N* = 59	Abnormal	*P*	Definition of abnormal
LV structure						
LVEDd (mm)	53.14 ± 7.67	25	49.45 ± 7.54	15	0.01	> 54 (men), > 50 (women)
LVESd (mm)	38.61 ± 9.52	30	35.30 ± 9.12	16	0.06	> 38.8 (men), > 35.5 (women)
IVSEd (mm)	12.66 ± 2.00	46	12.42 ± 1.91	40	0.5	> 11.5 (men), > 10.7 (women)
RWT	0.46 ± 0.09	41	0.49 ± 0.08	48	0.1	> 0.42
LVMI (g/m^2^)	165.70 ± 44.63	50	138.18 ± 44.69	44	< 0.001	> 115 (men), > 95 (women)
LV geometry					0.5	
Normal n (%)	2 (3.39)		2 (3.39)		—	—
Concentric remodeling n (%)	6 (10.17)		11 (18.64)		—	—
Concentric hypertrophy n (%)	37 (62.71)		31 (52.54)		—	—
Eccentric hypertrophy n (%)	14 (23.73)		15 (25.42)		—	—
LV systolic function						< 52 (men), 53 (women)
LVEF (%)	57.89 ± 9.35		58.00 ± 11.82		0.9	
≥ 57		38		42		
50–57		9		6		
40–49		12		11		
LVGLS	-14.87 ± 4.40	53	-16.91 ± 2.64	45	0.03	<-20%
LV diastolic function						
E/e′	17.19 ± 8.74		12.31 ± 6.42		0.002	> 14
>14		28		16	0.04	
≤14		31		43		
TRPV (m/s)	260.19 ± 37.19	24	254.73 ± 39.11	19	0.2	> 2.8
LA size and function						
LAV (ml)	66.54 ± 24.68	36	58.33 ± 23.94	26	0.08	> 58 (men), > 52 (women)
LAVI (ml/m ^2^)	37.69 ± 14.31		33.70 ± 13.40		0.1	> 34
>34		33		23	0.05	
≤34		26		36		
Valvular calcification		21		22	0.8	
MV		3		4		
AV		18		18		
Pulmonary pressure and right ventricle						
TAPSE (mm)	20.51 ± 4.12	7	20.12 ± 4.39	10	0.5	< 16
FAC (%)	46.72 ± 7.11	3	44.69 ± 8.03	5	0.3	< 35
PAsP (mmHg)	34.06 ± 9.75	31	31.57 ± 9.82	30	0.5	> 30
RIMP	0.49 ± 0.15	14	0.52 ± 0.13	21	0.2	> 0.55
RVGLS (%)	26.83 ± 6.82	17	27.45 ± 5.40	12	0.4	<-21
TAPSE/PAsP	0.69 ± 0.23		0.69 ± 0.25		0.8	
RVGLS/PAsP	0.84 ± 0.36		0.98 ± 0.29		0.1	

Values are expressed as n, mean ± SD, or n (%), unless specifically indicated

Abbreviations: SV, sacubitril valsartan; LVEDd, left ventricular end-diastolic diameters; IVSEd, interventricular septum end-diastolic thickness; RWT, relative wall thickness; LVMI, left ventricular mass index; LV, left ventricular; LVEF, left ventricular ejection fraction; LVGLS, left ventricular global longitudinal strain; E/e′, peak early diastolic transmitral flow velocity/peak early diastolic mitral annular tissue velocity; TRPV, tricuspid regurgitation peak velocity; LA, left atrial volume; LAV; left atrial volume; LAVI, left atrial volume index; MV, mitral valvula; AV, aortic valvula; TAPSE, tricuspid annular plane systolic excursion; FAC, fractional area change; PASP, pulmonary artery systolic pressure; RIMP, right ventricular myocardial work index; RVGLS, right ventricular global longitudinal strain

### Changes in typical echocardiographic parameters at baseline and 12 months later

**Fig. 2:**
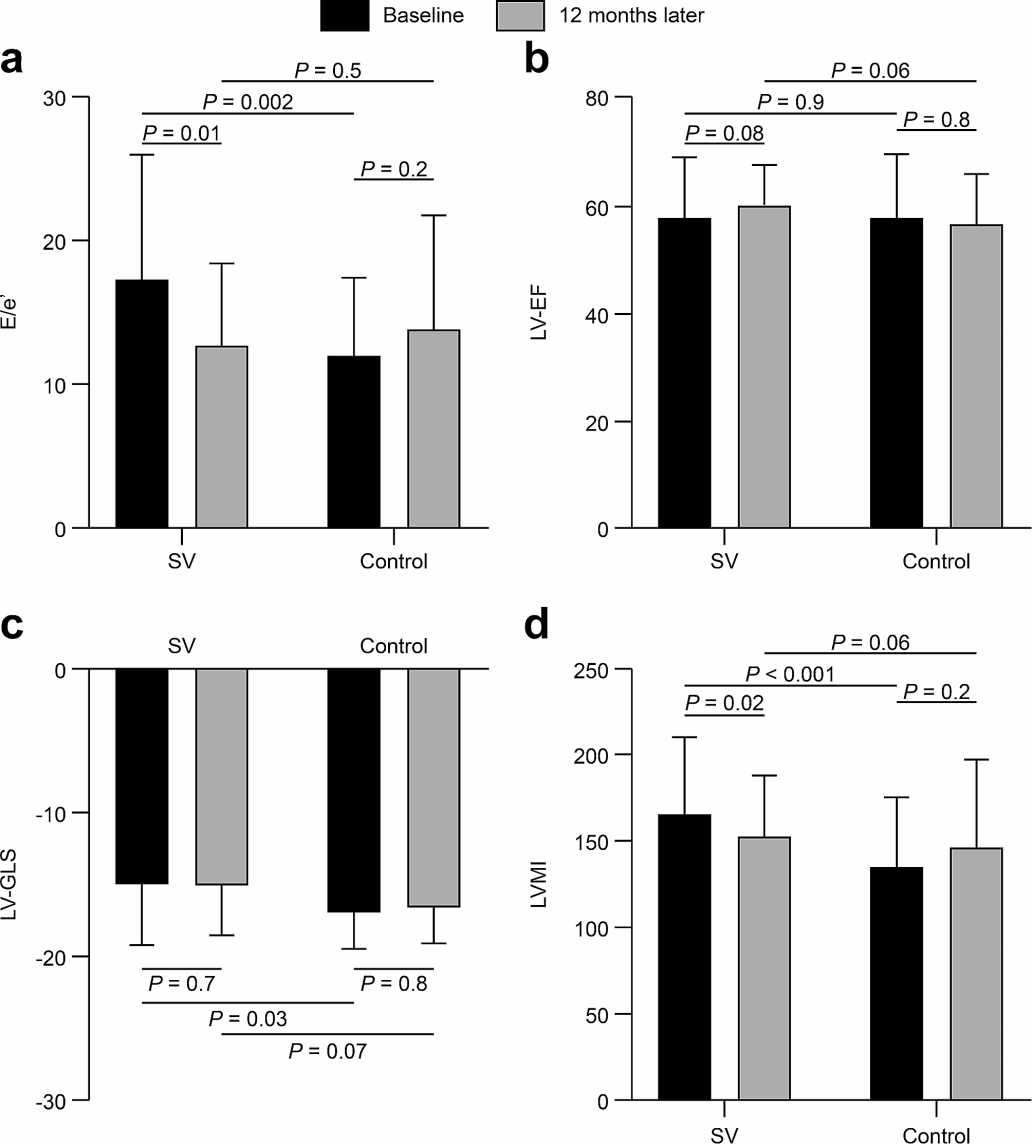
Cardiac structure and function at baseline and after 12 months in the SV and control groups Abbreviations: SV, sacubitril valsartan; LVMI, left ventricular mass index; E/e′, peak early diastolic transmitral flow velocity/peak early diastolic mitral annular tissue velocity; LVEF, left ventricular ejection fraction; LVGLS, left ventricular global longitudinal strain

The changes in typical echocardiographic parameters at baseline and 12 months later are shown in Fig. 2. After 12 months of SV treatment, E/e′ improved significantly (17.19 ± 8.74 vs. 12.80 ± 5.52, *P* = 0.01), and LVMI decreased from 165.7 ± 44.6 g/m^2^ to 154.8 ± 24.0 g/m^2^ (*P* = 0.02). LVEDd decreased from 53.14 ± 7.67 mm to 51.56 ± 7.44 mm (*P* = 0.03). The remaining parameters, including LVEF (*P* = 0.08) and LVGLS (*P* = 0.7), did not change significantly. Although LVGLS was lower in the SV group than in the control group at baseline (*P* = 0.03), the difference between the two groups was not significant after 12 months (15.24 ± 3.30 vs. 16.73 ± 2.41, *P* = 0.07). There were no significant differences in the echocardiographic parameters between baseline and 12 months later in the control group.

### Primary and secondary endpoints in both groups

During the 12 months of follow-up, there were 19 composite primary endpoint events in the SV group, including 17 HF hospitalizations and 2 cardiovascular disease-related deaths. In the control group, there were 24 composite primary endpoint events, including 20 HF hospitalizations and 4 cardiovascular disease-related deaths. The differences between groups were not statistically significant (*P* > 0.05).

Regarding the secondary endpoint, the ADQI class for HF improved in 39 patients and worsened for only 1 patient in the SV group, compared with 15 and 11 patients, respectively, in the control group. The difference was significant (*P* < 0.001) (Table 3).

**Table 3:** Primary and secondary endpoints for the sacubitril valsartan and control groups

Outcome	SV (*n* = 59)	Control (*n* = 59)	*P*
Primary Composite Endpoints			
Total number of hospitalizations for HF and death from cardiovascular causes	19	24	0.1
Total number of hospitalizations for HF	17	20	0.5
Death from cardiovascular causes, number (%)	2 (3.39)	4 (6.78)	0.3
Secondary endpoints			
Changes in ADQI HF class from baseline to 12 months, number (%)			Pearson X^*2*^ = 24.191 *P* < 0.001
Improved	39 (66.10)	15 (25.42)	--
Unchanged	19 (32.21)	33 (55.93)	--
Worsened	1 (1.69)	11 (18.64)	--
Death from all causes, number (%)	3 (5.08)	4 (6.78)	0.5

Values are expressed as n or n (%), unless indicated otherwise

Abbreviations: SV, sacubitril valsartan; HF, heart failure; ADQI, The Acute Dialysis Quality Initiative Workgroup

### Multivariate Cox regression of predictive factors for HF hospitalizations among patients on MHD

Predictive factors for HF hospitalizations among patients on MHD are presented in Table 4. Data from 155 eligible patients on MHD were included in Cox analysis for the first HF hospitalization and cardiovascular disease-related death. Ninety-six patients who were not administered SV had a total of 26 HF hospitalizations, and 4 died of cardiovascular causes. Age and diabetes mellitus, but not SV, were independent risk factors for HF hospitalization in both univariate and multivariate analyses (hazard ratio [HR] = 1.038, 95% confidence interval [CI], 1.017–1.059, *P* = 0.01; HR = 1.690, 95% CI, 1.012–3.570, *P* = 0.03). For echocardiographic parameters, only PAsP was an independent risk factor (HR = 1.771, 95% CI, 1.233–2.543; *P* = 0.002) on multivariate Cox analysis.

**Table 4:** Multivariate Cox regression model of risk factors for HF hospitalizations in maintenance hemodialysis patients (*n* = 155)

Factors	Univariate analysis				Multivariate analysis		
	HR	95% CI	*P*		HR	95% CI	*P*
Age	1.046	1.026–1.066	< 0.001		1.038	1.017–1.059	0.01
SV	0.810	0.389–1.683	0.6		0.694	0.316–1.567	0.2
DM	2.115	1.089–4.326	0.02		1.690	1.012–3.570	0.03
PAsP	2.019	1.420–2.870	< 0.001		1.771	1.233–2.543	0.002

Abbreviations: HR, hazards ratio; CI, credibility interval; SV, sacubitril valsartan; DM, diabetes mellitus; LVMI, left ventricular mass index; PAsP, pulmonary artery systolic pressure; HR estimates for continuous measures are per 10 mm Hg for PAsP

### Adverse events

During the 12 months of follow-up, there was no case of SV-related angioneurotic edema, severe hyperkalemia, and abnormal liver function. In 2 patients, the SV dose was maintained at 25 mg bid because of recurrent decreases in blood pressure (≤ 100/60 mmHg).

## Discussion

All the patients enrolled in the study had a hemodialysis vintage of 12 or more months and had entered the stable dialysis stage to minimize the effects of early dialysis on cardiovascular events [19, 20]. The N-terminal pro-B-type natriuretic peptide and high-sensitivity troponin are common biological markers for assessing HF in non-end stage renal disease patients; however, the value and diagnostic threshold in dialysis patients remain controversial [21]. Therefore, biological markers were not used as diagnostic and enrollment criteria for HF in the study. Clinical symptoms of HF and echocardiographic evidence of cardiac structural and functional abnormalities were set as diagnostic criteria. Heart function was evaluated using the ADQI class for HF and the Chinese guidelines for HF in dialysis patients [14, 21]. The primary and secondary endpoints in this study did not also include biological markers of HF.

There is clinical evidence of the usefulness of SV for the treatment of hypertension and the delay of residual renal function loss in patients with chronic kidney disease and reduction of HF hospitalizations and cardiovascular disease-related mortality rates in patients with HFrEF [7–9, 22]. For non-end-stage renal disease patients, the target dose of SV is 400 mg per day administered as 200 mg twice daily. However, the target dose for dialysis patients was 100 mg twice daily in most studies [9, 22, 23]. Considering that SV could not be cleared by dialysis [24], the dose range of SV for patients receiving MHD in this study was 50–200 mg/day.

This was a real-world study. Analysis of data of the enrolled patients showed more male patients and lower LVEF in the SV group than in the control group. Therefore, the two groups were matched at a ratio of 1:1 based on LVEF. There were no significant differences in the clinical characteristics at baseline. There were some differences in echocardiographic parameters, although LVEF was matched; LVMI, E/e′, and LVEDd were higher while LVGLS was lower in the SV group than in the control group. These results suggested that cardiac diastolic and systolic functions were more impaired in the SV group. LVEF is preserved in HFpEF; however, impairments in the LV structure and diastolic and systolic functions are distinguishing characteristics [25, 26].

The present study differs from previous studies [27–29] in terms of the value of echocardiographic parameters in predicting HF and cardiovascular events. After 12 months of SV treatment, the changes in LVEF and LVGLS were not significantly different, unlike LVMI, LVEDd, and E/e′. LVEF did not independently predict the risk of cardiovascular events in patients with HFpEF in the PARAGON study, whereas LVMI, LAVI, and E/e′, among others, were independent risk factors [26]. Various explanations were considered: previous clinical studies on HFpEF did not include patients on MHD [27–29], cardiovascular morbidity and mortality are higher in patients on MHD, and various factors can affect heart function in patients on MHD. The LVEF values in this study were close to those reported in previous studies [6, 26–29]; however, LVH and LVMI were higher in patients on MHD in this study. This may have led to bias and affected the results of the Cox analysis. The differences in echocardiographic parameters between the two groups at baseline and the results of the Cox analysis suggested a strong heterogeneity in the clinical presentation of HFpEF.

In this study, age and diabetes mellitus remained independent risk factors for HF hospitalization and cardiovascular disease-related mortality in patients on MHD. Moreover, as in previous studies [5, 6], SV did not result in improvements in HF hospitalization or cardiovascular disease-related mortality in patients on MHD with an EF above 40% during the 12 months of follow-up. However, SV significantly improved the secondary clinical outcome and ADQI class for HF in patients on MHD. Considering the improvements in LV structure and diastolic function, better outcomes are likely to be observed with a longer follow-up.

In addition to left HF, patients on MHD often have a combination of right HF and pulmonary hypertension. The incidence of pulmonary hypertension in patients on MHD ranged from 25 to 49% [30, 31]. In this study, more than 50% of patients on MHD with an LVEF of > 40% had PAsP of > 30 mmHg, and Cox analysis showed that PAsP was an independent risk factor for HF hospitalization and cardiovascular disease-related mortality, indicating the detrimental effect of pulmonary hypertension in patients on MHD with HFpEF. PAsP, RIMP, RVGLS, TAPSE, and FAC, which reflect right heart function, did not improve after SV treatment. SV has been shown to reduce pulmonary artery vessel wall thickness and improve right ventricular remodeling in animal experiments [32, 33]. A few articles have reported that SV improves pulmonary hypertension and right HF symptoms in patients with HFrEF (excluding patients with abnormal renal function) [34, 35]; however, they could not exclude the likelihood that improvement in right heart function resulted from the improvement in left heart function. Robust evidence on the effectiveness of SV in improving right HF is lacking.

This study has some limitations. It involved a small sample and was a single-center observation. In this real-world study, the two groups were matched at a ratio of 1:1 based on LVEF; some echocardiographic parameters, such as LVEDd, LVGLS, LVMI and E/e’, were still worse in the SV group than in the control group. However, to our knowledge, it is the first study to investigate the effects of SV on HF hospitalization and cardiovascular disease-related mortality in patients on MHD with HFpEF. The insights from this study provide directions for further studies exploring the reduction and prevention of HF hospitalization and cardiovascular disease-related mortality of MHD.

## Conclusions

This study initially showed that SV partially improved LV diastolic function and the ADQI class for HF in patients on MHD with an EF of > 40%. However, it failed to reduce the composite endpoints of HF hospitalization and cardiovascular disease-related mortality over 12 months of follow-up. Further clinical studies involving patients on MHD are expected in the future.

## Data Availability

The datasets used and/or analyzed during the current study are available from the corresponding author on reasonable request.

## Abbreviations

ADQI: Acute Dialysis Quality Initiative Workgroup
EF: Ejection fraction
FAC: Fractional area change
HF: Heart failure
HFmrEF: HF with mid-range EF
HFpEF: HF with preserved ejection fraction
HR: Hazard ratio
LVEDd: Left ventricular end-diastolic diameter
LVGLS: Left ventricular global longitudinal strain
LVMI: Left ventricular mass index
MHD: Maintenance hemodialysis
PASP: Systolic pulmonary artery pressure
RIMP: Right ventricular myocardial work index
RVGLS: Right ventricular global longitudinal strain
SV: Sacubitril valsartan
TAPSE: Tricuspid annular plane systolic excursion
