# Correction to: Effect of sacubitril valsartan on heart failure with mid-range or preserved ejection fraction in patients on maintenance hemodialysis: real-world experience in a single-center, prospective study

**DOI:** 10.1186/s12872-024-03801-6

**Published:** 2024-03-02

**Authors:** Xiao-mei Huang, Jing-jing Li, Wang Yin, Hui-ling Fu, Fen Yu, Lian-qing Gu, Yi Zhang, Min Du, Zheng Ye, Li Xu

**Affiliations:** 1 Department of Nephrology, Tongji Medical College, The Central Hospital of Wuhan, Huazhong University of Science and Technology, Wuhan, 430014 China; 2 Department of Ultrasound, Tongji Medical College, The Central Hospital of Wuhan, Huazhong University of Science and Technology, Wuhan, 430014 China; 3 Department of Public Health, Tongji Medical College, The Central Hospital of Wuhan, Huazhong University of Science and Technology, Wuhan, 430014 China

**Correction to: BMC Cardiovascular Disorders (2024) 24:1**

10.1186/s12872-024-03744-y.

Following publication of the original article [1], author Li Xu also should have been denoted as a corresponding author.

The original article has been corrected.

## References

[1] Huang X, Li J, Yin W (2024). Effect of sacubitril valsartan on heart failure with mid-range or preserved ejection fraction in patients on maintenance hemodialysis: real-world experience in a single-center, prospective study. BMC Cardiovasc Disord.

